# Jottings

**Published:** 1909-12-11

**Authors:** 


### Jottings.

The Duchess of Albany was present on December 2 at the jubilee entertainment of the National Hospital for the Paralysed and Epileptic. It was held for the special benefit of the patients, nurses, and household staff.

The Southgate District Council, after much consideration, have decided to contribute to the Wood Green, Hornsey, and Southgate Hospital £1 for each Southgate resident treated as an in-patient, except in cases where a guinea a week or more is paid privately on the patient's behalf.

The fifty-fifth annual meeting and the one hundred and eleventh half-yearly election of the Putney Royal Hospital for Incurables was held on Friday, November 26, 1909, at the Cannon Street Hotel, E.C. Thirty candidates were elected.

The "Falmouth Booklet" is an interesting, useful, and handsomely got-up illustrated guide to this favourite resort of the Cornish Riviera, which has just been issued by the Corporation. It can be obtained on application to the London Information Office, 29 John Street, Bedford Row, W.C.

Mr. James Boyd presided at the annual festival dinner in aid of the West Ham and East London Hospital on December 3. Donations and subscriptions amounting to about £2,000, including the Chairman's list of £1,500, were announced. As regards the extension scheme, a new operating theatre, out-patients' department, and dispensary are already in use, and it is hoped that the whole of the buildings will be completed early next year.

At the annual meeting of the Greenock Eye Infirmary it was reported that a serious epidemic of purulent ophthalmia had broken out at the beginning of July last, but no serious defects of vision had resulted. It is difficult to state the exact origin of the epidemic, but the want of cleanliness and badly ventilated houses were the probable causes. Indoor patients to the number of 117 have been admitted during the year, and the average residence was 13 days.

Lady Constance Hatch opened a bazaar at the Royal Ear Hospital, Soho, on December 2.

The fifty-fourth annual festival dinner of the Poplar Hospital for Accidents, Poplar, E., will be held at the Holborn Restaurant, High Holborn, on Tuesday, December 14, 1909, at 7 p.m. precisely. The Right Hon. Sir Hudson E. Kearley, Bart., M.P. (Chairman of the Port of London Authority), in the chair.

At the annual meeting of the Royal Hospital for Incurables on November 26 the Chairman said that their income had exceeded their expenditure, but this was largely owing to legacies, and as the result of his appeal the Duchess of Northumberland had sent them a full-size billiard table for the men's wing.

Sir Hudson E. Kearley, M.P., will preside at the Poplar Hospital annual dinner at the Holborn Restaurant on December 14. The following gentlemen have accepted the office of vice-president of the hospital: Mr. Sydney Buxton, M.P. (Postmaster-General); Mr. C. J. Cater Scott, Mr. F. J. Mirrielees, Mr. Edward F. Turner, and Dr. Edwin Freshfield.

On the motion of Sir Charles Seely, Bart., seconded by Mr. T. L. K. Edge, the following resolution was passed at the last monthly meeting of the Board of the Nottingham General Hospital: "That the monthly board have heard with deep regret of the death of Mr. Thomas Hill, who for many years was closely connected with the hospital, and desire to express their sincere sympathy with the members of his family in their bereavement."

Princess Henry of Battenberg visited Camelford House, Hereford Gardens, Marble Arch, on December 2 to open a three-days' sale of work on behalf of the General Lying-in Hospital, York Road, Lambeth. Her Royal Highness was received by the Duchess of Buccleuch (Chairman) and other members of the General Committee. It is hoped that the bazaar will realise at least £1,000, which will enable some essential repairs and decoration to be carried out.

